# Latest Advances in Highly Efficient Dye-Based Photoinitiating Systems for Radical Polymerization

**DOI:** 10.3390/polym15051148

**Published:** 2023-02-24

**Authors:** Alicja Balcerak, Janina Kabatc-Borcz, Zbigniew Czech, Marcin Bartkowiak

**Affiliations:** 1 Department of Organic Chemistry, Faculty of Chemical Technology and Engineering, Bydgoszcz University of Science and Technology, Seminaryjna 3, 85-326 Bydgoszcz, Poland; 2 International Laboratory of Adhesives and Self-Adhesive Materials, Department of Chemical Organic Technology and Polymeric Materials, Faculty of Chemical Technology and Engineering, West Pomeranian University of Technology, Pułaskiego 10, 70-322 Szczecin, Poland

**Keywords:** squaraines, pyrrole derivatives, BODIPYs, sensitizers, dyeing photoinitiating systems, light-induced radical polymerization

## Abstract

Light-activated polymerization is one of the most important and powerful strategies for fabrication of various types of advanced polymer materials. Because of many advantages, such as economy, efficiency, energy saving and being environmentally friendly, etc., photopolymerization is commonly used in different fields of science and technology. Generally, the initiation of polymerization reactions requires not only light energy but also the presence of a suitable photoinitiator (PI) in the photocurable composition. In recent years, dye-based photoinitiating systems have revolutionized and conquered the global market of innovative PIs. Since then, numerous photoinitiators for radical polymerization containing different organic dyes as light absorbers have been proposed. However, despite the large number of initiators designed, this topic is still relevant today. The interest towards dye-based photoinitiating systems continues to gain in importance, which is related to the need for new initiators capable of effectively initiating chain reactions under mild conditions. In this paper we present the most important information about photoinitiated radical polymerization. We describe the main directions for the application of this technique in various areas. Attention is mainly focused on the review of high-performance radical photoinitiators containing different sensitizers. Moreover, we present our latest achievements in the field of modern dye-based photoinitiating systems for the radical polymerization of acrylates.

## 1. Introduction

Over the last few decades, dynamic developments within photochemistry have contributed to a better knowledge of photopolymerization processes. As is well known, the energy provided by light is readily available and is a good alternative for starting the chemical reactions. For this reason, light-based processes hold a great promise for a greener future and sustainable development [1]. Numerous studies on photochemistry, organic chemistry and polymer chemistry have given rise to a new and innovative production method for various synthetic materials. Therefore, light-induced polymerization reactions have become an interesting area for academic groups and industrial researchers. Moreover, photoinduced polymerization is considered one of the key technologies of Industry 4.0 [2,3,4].

Photopolymerization shows a range of advantages over thermally initiated processes. It can be concluded that this technique perfectly reflects the principle of “more for less” [5]. This is due to lower economical costs [6], lower energy consumption [7], fast reaction rate [8], mild reaction conditions [9], possibility of temporal and spatial control of process [10], reduced organic solvents consumption, toxic substances and post-production waste [11], etc. This strategy for the synthesis of polymer materials has given a significant boost to the development of modern technologies using light as an energy source. The possibilities offered by photopolymerization are enormous, which extends to a wide range of applications [12].

**Figure 1:**
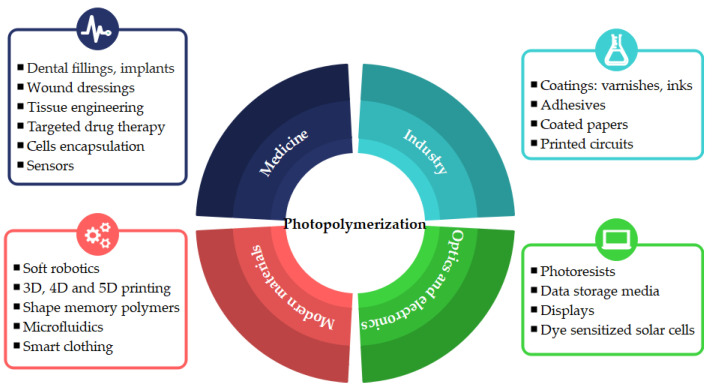
Areas with applications for photopolymerization technology.

Photopolymer materials are used in many fields of science and technology [13]. There are four main directions of application of photopolymerization processes, i.e., medicine [14], the manufacturing industry [15], optics and electronics [16] and advanced materials and modern technologies [17]. The basic application areas are presented in Figure 1.

As presented in Figure 1, one of the most important areas of application is medicine. Major segments in this sector include stomatology, new therapies for the treatment of various diseases, 3D imaging, dressing materials and medical devices [18]. Photopolymerization is the major reaction used for the filling of dental cavities and restoration of teeth. The replacement of the previously used unsightly and potentially harmful amalgam fillings with light-cured resin-based composites (RBCs) was a significant step towards the development of modern dentistry. Novel dental fillings show better esthetics and good mechanical properties, therefore becoming basic materials for the reconstruction of the hard tissues of the tooth [19,20,21]. Another important aspect is the design and fabrication of various types of wound dressings and advanced medical devices. Photopolymers perfectly meet the fundamental criteria for medical applications, such as biocompatibility, non-toxicity, biodegradability and, in some cases, the ability to self-regenerate. For this reason, these materials are used in tissue engineering, regenerative biomedicine, drug delivery systems, vaccine adjuvants and the production of hydrogels, among other uses [18,22,23].

In the chemical industry, photoinitiated polymerization reactions are relevant most of all for the manufacture of a wide range of coatings, e.g., lacquers, sprays, inks and adhesives. These coatings protect the target products against corrosion, scratching, different chemical and biological substances, wear or decomposition and ensure the esthetic appearance of the coated surface [24,25,26]. Dynamic developments of coating production technology are reflected directly by numerous inventions in the field of new polymer products. Nano- and fluoropolymer coatings are a few examples of materials that exhibit remarkable properties, such as long life cycle and high resistance to destructive biological and physicochemical factors. According to the latest reports, it is estimated that photocurable coatings’ global market will reach USD 10,751.64 million by 2026. The key factors responsible for the growth of production include the growth of automotive and construction industries, as well as increasing demand for inks and coatings for the packaging industry. The numerical data shows how large the market is for protective, decorative and adhesive coatings [27,28,29,30].

It should be mentioned that optoelectronics also owes its progress to the use of materials produced by light-induced polymerization. Photopolymers have become a versatile and attractive material for holographic recording because of their relatively low cost, ease of manufacturing, flexibility of shape and self-developing character [31,32].

Better knowledge of photopolymerization processes has allowed for increasing the scale of production for new polymers. Indeed, the quick development of 3D printing techniques has also contributed to the introducing the most significant improvements in various research areas [33]. In 2022, many promising inventions have been reported. For example, Wang and others designed innovative artificial conduits providing protection against postoperative infections and acute thrombosis [34]. In the same year, Dey and colleagues described the possibility of using post-production wastes in 3D concrete-printing processes [35]. Equally surprising is the research paper that shows the potential of a 3D technique in the fabrication of hydrogel composites [36].

It is worth paying attention to the latest generation of materials, known as “smart materials”, which may revolutionize the future and conquer the world [37]. There are different smart materials which change specific properties as a result of the action of an appropriate external stimulus. This group includes electroactive, magnetostrictive, piezoelectric, ferroelectric, biomimetic, thermo-responsive and chromic materials, shape-memory materials, aerogels, optical fibers, quantum-tunneling composites, self-healing and electroluminescent materials, etc. [38]. It should be noted that many materials have already proved their practical effectiveness; for example, shape-memory polymers [39,40], self-repairing coatings [41,42], smart glass [43], graphite fibers [44], self-healing polymeric coatings [45] and many others [38].

The examples discussed present only a small part of the application fields for photopolymerization processes. The potential of this technique is powerful and promising, and the development opportunities for the production technologies of functional photopolymer materials are much greater. Hence, the further study of the field of photochemistry and polymer chemistry is quite reasonable.

## 2. Photoinitiated Radical Polymerization

### 2.1. Mechanism of Photopolymerization

**Scheme 1:**
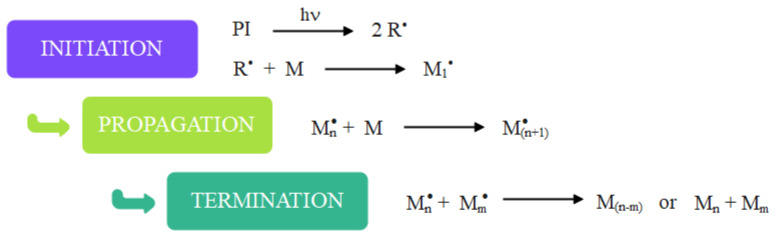
Major steps of radical polymerization process.

Photopolymerization is a process in which a light source provides the energy necessary to initiate a chain reaction and cause a rapid conversion of liquid monomer/oligomer into solid linear or crosslinked polymer [46]. The activation of photopolymerization requires not only light energy but also the presence of a suitable initiator. This compound is a driving source of the polymerization process because it enables the formation of initiating radicals. Therefore, it can be concluded that these two factors are decisive for efficient photopolymerization. In general, radical polymerization includes three fundamental steps—initiation, propagation and termination—which are presented in Scheme 1 [47].

The first stage of polymerization involves two basic reactions, i.e., (i) primary radical formation (R^•^) and (ii) addition of reactive species (R^•^) to the first monomer molecule (M), which leads to the production of chain-initiating radicals (M_1_^•^). This clearly indicates that the initiation efficiency depends substantially on the type of photoinitiator (PI) in a polymerizable mixture. Therefore, the decomposition of an initiator triggered by light action determines the rate of this process. The propagation reaction describes the addition of successive radical species (M_n_^•^) to the monomer, which results in the growing of macromolecules (M_(n+1)_^•^). The final step, called termination, interrupt the growth of macromolecules. It is related to the low concentration of the active centers, about 10^−8^ mol × dm^−3^, which limits the possibility of further growth of the polymer chain. Generally, there are two major pathways toward ending a propagation step: recombination and disproportionation. In the first case, the macroradicals react with each other forming a single, nonreactive chain. There is also a possibility of the combination of a growing end of a polymer chain with a free radical. The termination by disproportionation includes the abstraction of a hydrogen atom by one of the propagating units, forming another chain. As a result, two terminated chains (one unsaturated and the other saturated) are formed. The selectivity of recombination and disproportionation reactions affects the molecular weight and the structure of polymer and, thus, the properties of the final product [47,48,49,50,51,52].

### 2.2. Types of Photoinitiators

As mentioned in the previous section, a precondition of the initiation of the polymerization process is the presence of an appropriate initiator in the polymerizable composition [53]. In general, there are two main classes of photoinitiators: radical and cationic. Radical initiators can react diversely upon UV/Vis radiation. Depending on the pathway of active species formation, these compounds are classified as type I and type II PIs [54,55].

**Scheme 2:**
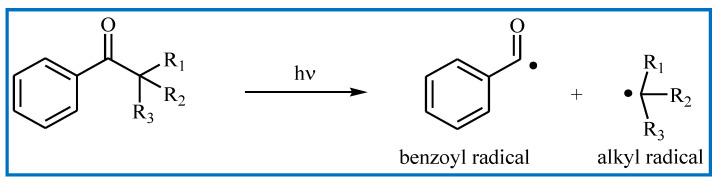
Photoinitiation of polymerization process using type I photoinitiator.

Type I photoinitiators are unimolecular initiators, which undergo a homolytic or heterolytic bond cleavage after light excitation forming two initiating centers. Depending on the type of mechanism of generation of active radicals, photoinitiators can be divided into two groups, namely, α- and β-cleavable. Definitely, the most commonly used compounds are α-cleavable Norrish type I initiators. In this case, the photolysis occurs at the carbonyl alpha atom on the weak C=O-alkyl bond. On the other hand, the presence of a weak bond in the beta-position, e.g., C-Cl, C-N or C-S, makes it susceptible to cleavage at this place. The general mechanism of generating initiating radicals in photoinitiating systems containing α-cleavable PI (benzoin) is presented in Scheme 2 [55,56,57].

The type I initiators are characterized by high values of the cleavage rate constant. Importantly, this feature is crucial for high quantum yield of radical generation (Φ_R_) and, thus, enables effective initiation of polymerization. The vast majority of compounds classified as type I PIs show values of Φ_R_ in the range of 0.8–1. However, the main drawback of photoinitiating systems containing α-cleavable initiators is their limited spectral sensitivity, including mainly the ultraviolet region of light.

The worldwide market offers a large range of different types of initiators. Among type I photoinitiators, the most used compounds are [58]:

- Acylphosphine oxides (APOs);
- Benzil ketals (BKs);
- Benzoin ethers (BEs);
- α-Hydroxyalkyl ketones (HPs);
- Mixtures of the above-mentioned compounds (blends) and others.

**Figure 2:**
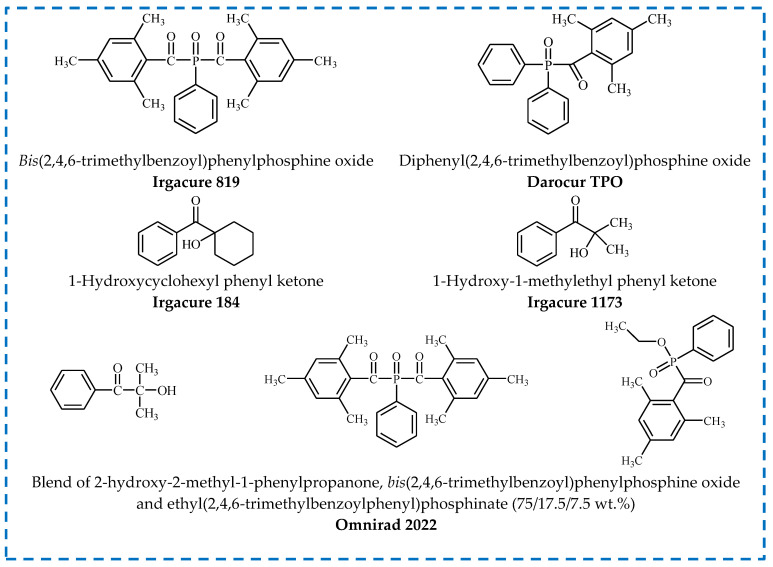
Examples of commonly used type I photoinitiators.

The structures of several, commonly used type I PIs are shown in Figure 2 [58,59,60,61].

The compounds belonging to the group of acylphosphine oxides (APOs) have a narrow absorption band in the long UV region, which makes them perfect to depth cure. Moreover, the non-yellowing and photobleaching properties enable the use of these initiators in thick and highly pigmented compositions. On the other hand, benzoin ethers (BEs) may cause yellowing of the coating, but show high performance for curing of unsaturated polyester/styrene formulations. For example, the α-hydroxyalkyl ketones (HPs) are mainly used in clear coatings, overprint lacquers, adhesives, topcoats for plastics, metals, wood, etc. In addition, systems containing more than one initiator (known as blends) are also available on the market. For example, commercial photoinitiator Omnirad 2022 is a highly efficient curing agent used for initiation of radical polymerization of unsaturated resins based on the prepolymers [55,62,63,64,65].

**Scheme 3:**
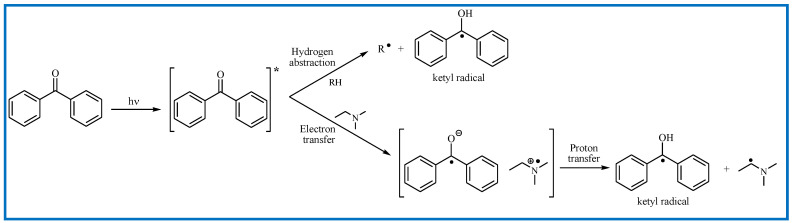
Photoinitiation of polymerization process using type II photoinitiator.

Type II photoinitiators are combinations of two or more components which form relatively long-lived excited triplet states capable of hydrogen abstraction or electron/proton transfer processes with other molecules, called as co-initiators. Therefore these initiators are not suitable for use with formulations comprising strong triplet quenchers. There are two basic routes of formation for initiating species. The major mechanism of photoinitiation of polymerization reactions in the presence of a type II photoinitiator is shown in Scheme 3 [62,65].

One of the possible directions of reactions is based on hydrogen abstraction by the long-lived n-π^*^ excited triplet state from the donor molecule. As a result, a very active donor radical is formed. The donors contain heteroatoms with active hydrogen atoms in the α-position, e.g., alcohols, ethers, esters, tertiary amines, thiols and others. If the donor is tertiary amine, the primary reaction is an electron transfer process (ET) from the nitrogen atom to an excited state of an electron acceptor, which leads to the formation of a radical ion pair: radical cation/radical anion. Then, the proton transfer process from α-carbon atom to the donor leads to the formation of two radicals: ketyl and α-aminoalkyl [62,65,66].

In contrast to the type I photoinitiators, the performance of type II initiators depends not only on their physico-chemical properties but also on the type of co-initiator used. It is considered that type II photoinitiators may be less effective due to bimolecular processes, back electron transfer and the solvent cage effect in solutions. However, despite minor difficulties, these compounds work well as highly active initiators of polymerization reactions.

Among type II initiators, the most popular are [58]:

- Anthraquinones (AQs);
- Benzophenones (BPs) and their substituted analogues;
- Camphorquinones (CQs);
- Thioxanthones (TXs);
- Mixtures of the above-mentioned compounds (blends) and others.

**Figure 3:**
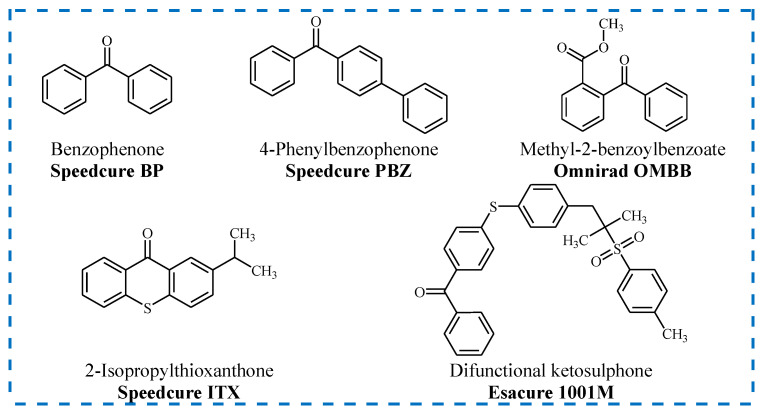
Examples of commonly used type II photoinitiators.

Examples of type II initiators used in radical polymerization reactions are presented in Figure 3 [62,67].

One of the best known type II PIs is benzophenone and its derivatives. Importantly, an appropriate modification of the structure of BP results in the extension of the absorption band to the visible region of the spectrum and an improvement in reactivity. Other significant groups of photoinitiators include camphorquinones and thioxanthones. For example, Speedcure ITX is a high-performance initiator which, in combination with an amine synergist, induces rapid photopolymerization reactions. The most important advantage of this PI is good solubility in most popular organic solvents and monomers as well as intensive absorption. On the other hand, difunctional ketosulphone—known also under commercial trade as Esacure 1001 M—is suitable for all systems where low migration is needed. Moreover, such features as high reactivity, minimal post-curing odor and very low volatile organic compound emission (VOCs) makes it appropriate for use not only in the chemical industry but also in food packaging [62,68].

In conclusion, regardless of the type of mechanism of reaction, the presence of a photoinitiator is necessary to initiate the photoinduced radical polymerization process.

## 3. Dyes as Effective Sensitizers in Photoinitiating Systems

**Figure 4:**
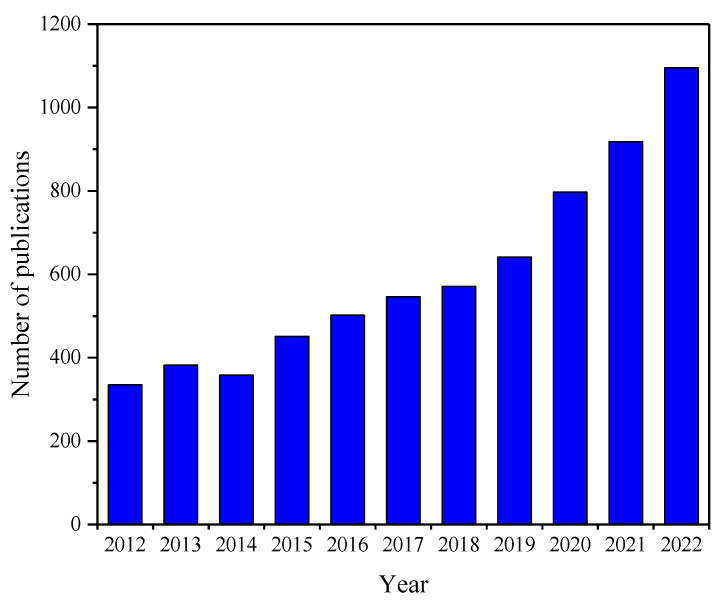
The number of research papers published per year focused on photoinitiating systems for photopolymerization according to Science Direct. Keyword: photoinitiator. Accessed on 13 February 2023.

Over the years, numerous color compounds have been synthesized, studied and described by diverse research groups. Nevertheless, the need for various types of dyes is still enormous due to their applicability in various fields of science and technology [69]. Apart from the basic use of these compounds, which is the coloring of different types of materials, another important area is the use as sensitizers in photopolymerization reactions. The interest in the design of new photoinitiators, including photoinitiating systems based on dyes, is huge, as evidenced by the numerous papers published in this field. The data analysis of numbers of publications on the discussed topic with regard to the scientific community clearly indicate that, since 2012, a total of 6964 research works focused on photoinitiators were published. It should be noted that a pronounced increase of the number of published documents in every year is observed, as is shown in Figure 4.

**Figure 5:**
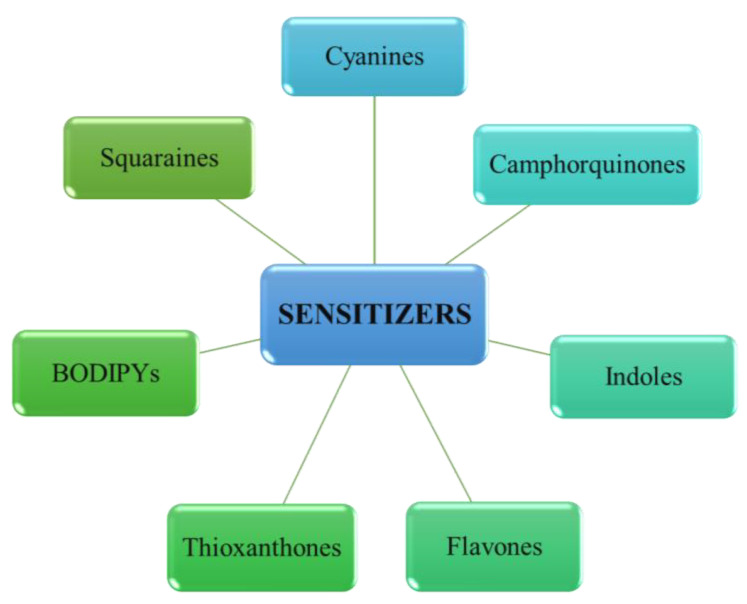
Popular sensitizers used in photoinitiating systems.

Many various organic dyes as photosensitizers for radical, cationic and hybrid polymerization of various monomers have been described in the literature so far. These compounds show different absorption profiles, thus operating over a wide spectral range from ultraviolet (UV) to visible light (Vis). Examples of selected color compounds used as light absorbers in photoinitiating systems are shown in Figure 5 [70].

Crucial for light-activated polymerization, of concern for photoinitiating systems, are compounds that allow for light energy conversion into the appropriate chemical energy for reactive intermediates [71]. Due to the number of components included in the PIS, the following systems can be distinguished: (i) one-component, (ii) two-component and (iii) multi-component. One of the most popular are bimolecular photoinitiating systems, containing a dye molecule acting as sensitizer and a second compound which plays the role of co-initiator. These PISs generate radicals, usually as a result of photoinduced electron transfer processes (PET) between sensitizer and co-initiator [72].

**Figure 6:**
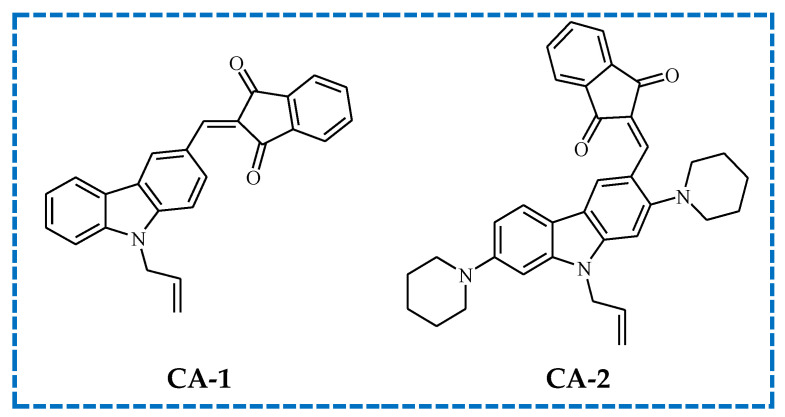
Chemical structures of carbazole-indanedione sensitizers proposed by Liao and colleagues [72].

One of the latest developments in the design of dye-based photoinitiating systems are carbazole-indanedione-based photoinitiators described by Liao and co-workers. The group of scientists presented the sensitizing abilities of novel dyes (chemical structures of these dyes are presented in Figure 6), which show absorption in the UV-Vis range.

The best results were obtained for PIS containing CA-2 dye. This sensitizer in combination with iodonium salt (diphenyliodonium hexafluorophosphate) provides highly efficient photoinitiation for hybrid polymerization of an acrylate/epoxyacrylate mixture. The maximum values of monomer conversion equal to 50% (for 1,6-hexanediol diacrylate, HDDA) and even from 40% to 50% (for epoxide/acrylate blend in laminate conditions) were achieved. Importantly, the proposed initiators showed high photobleaching properties, which may have a huge potential for deep curing of polymerizable compositions [73].

**Figure 7:**
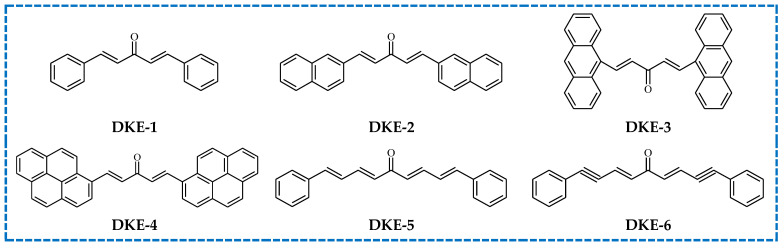
Chemical structures of *bis*-chalcone sensitizers proposed by Deng and Qu [73].

Other promising candidates include photoinitiators based on the *bis*-chalcone structure (structural formula of dyes are shown in Figure 7).

These compounds—proposed by Deng and Qu—are characterized by a wide range of absorption, from 300 nm to 500 nm, and quite high molar absorption coefficients, about 5 × 10^4^ M^−1^ × cm^−1^. The photopolymerization experiments were carried out for three-component systems consisting of an appropriate *bis*-chalcone sensitizer (DKE-1–DKE-6), diphenyliodonium hexafluorophosphate (Iod) and triethanoloamine (TEOA). The highest kinetics parameters of polymerization of poly(ethylene glycol) diacrylate (PEGDA) under 405 nm LED were obtained for compositions containing 0.1 wt% DKE, 2.0 wt% Iod and 2.0 wt% of TEOA. For these systems, the values of final monomer conversion ranged from 60% to ca. 90% for the most effective PI. Besides high conversion rates, newly synthesized initiators show good solubility in various monomers and a low migration ratio. Due to low toxicity, the proposed photoinitiating systems can be successfully used in the medical sector, e.g., as initiators of polymerization of light-cured dental compositions [74].

**Figure 8:**
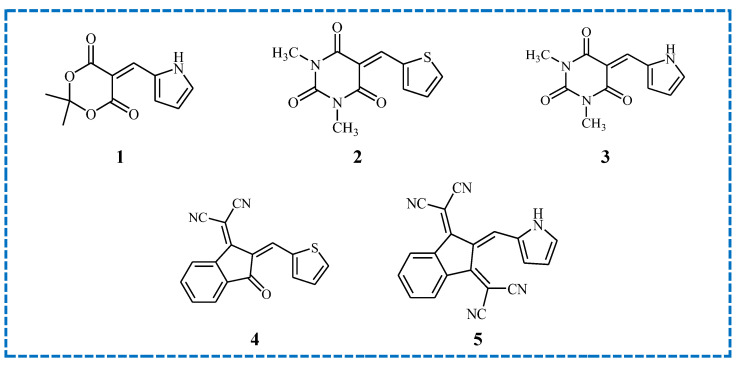
Chemical structures of push–pull sensitizers proposed by Sun and colleagues [75].

An interesting group of sensitizers of polymerization reactions are push–pull dyes. These compounds are composed of electron-donating and an electron-accepting moieties connected by means of a π-conjugated spacer. Indeed, this chemical structure provides the opportunity to finely tune absorption spectra, which is important for the easier matching of sensitizer to the light source used. Taking into account that push–pull dyes exhibit high molar extinction coefficients (ε), these compounds are suitable candidates for practical applications, e.g., in optoelectronics [75,76]. The use of this type of dye in three-component photoinitiating systems was described in 2021. The push–pull dyes were combined with two different co-initiators, such as an iodonium salt (Speedcure 938) and a tertiary amine (ethyl dimethylaminobenzoate, EDB), and examined for photosensitization capabilities of the polymerization reaction of tetrafunctional polyether acrylate (TA). The proposed sensitizers (chemical structures are presented in Figure 8) showed high light-absorption properties from 320 nm to even 600 nm. The irradiation of a polymerizable mixture with LED light (wavelength: 405 nm) resulted in high monomer conversion values above 90%. Importantly, the described initiators are characterized by short induction time of polymerization, whereby the curing of compositions occurred within 60–400 s. Surprisingly, some sensitizers in the presence of iodonium salt and amine also initiated polymerization processes with sunlight [76].

It is worth mentioning the useful of organic chromophores in self-healing processes, which are crucial for the production of new-generation materials, especially smart coatings and polymers [77,78]. The connection of light as an external stimulus and appropriate photoinitiating system may be a good direction for the regeneration of various materials. For example, self-healing with the use of microcapsules is a promising method to enhance the life of polymeric coatings [79]. As was described by Song and others [80], self-healing systems containing fluorescent-dye-loaded microcapsules can be used as high-performance agents for repairing epoxide resin. The chromophores used showed yellowish fluorescence in cracked regions and greenish fluorescence in healed regions, which enabled monitoring of the condition of the emerging defects in the light-cured layer. Recently, an interesting paper focused on clearcoat for the automotive industry was published [81]. The research group developed self-healing lacquer with a reversible polymer network composed of acryl polyol, dynamic hindered urea and diammonium borate dye. In this case, self-regeneration of the scratched coating was photothermally triggered and was based on dissociation and recombination of network structures. The proposed clearcoat showed rapid healing (about 100% efficiency of self-healing after ~30 s) even under sunlight. This lacquer can be a great alternative for typical commercially available automotive coatings [81].

Substantially, the organic dyes can also act as activators for reversible deactivation radical polymerization (RDRP). Developing highly efficient photocatalysts (PCs) is crucial for direct conversion of light energy into chemical transformations. The photoredox catalysts based on chromophores play important roles in photoinduced electron/energy transfer–reversible addition–fragmentation chain transfer polymerization (PET-RAFT) and metal-free atom transfer radical polymerization (ATRP) [82,83,84]. The characteristic feature of reversible deactivation radical polymerization is a rapid initiation step and reduction of irreversible termination reactions. The effective minimization of radical termination extends the lifetime of the propagation step and allows for the control of the macromolecular composition, molecular weight, structure and length of a polymer chain. Numerous works in redox photocatalysis have contributed to the development of RDRP in the production of versatile and well-defined polymer structures [85,86].

Detailed information on PET-RAFT processes can be found in the literature [87,88,89]. It should be highlighted that there are many uncertainties about the mechanism of controlled reversible radical polymerization processes based on the electron/energy transfer reactions. Generally, two main routes of PET-RAFT are proposed. The originally suggested pathway assumes the photoinduced electron transfer from the excited state of a photocatalyst (PC) to the thiocarbonylthio-containing molecule (known also as RAFT agent or chain transfer agent, CTA). In the next step, the fragmentation of this species leads to the generation of anionic CTA adduct and radicals. The radicals can undergo monomer addition and also interact with the reduced chain transfer agent and the oxidized PC, closing the catalytic cycle. On the other hand, an alternative pathway refers to the photoinduced energy transfer. This mechanism is assigned to Dexter electron exchange, which occurs between the photocatalyst and RAFT agent. The excited chain transfer species undergoes fragmentation, giving CTA radicals and radical polymerization-initiating forms [88,89,90,91].

**Figure 9:**
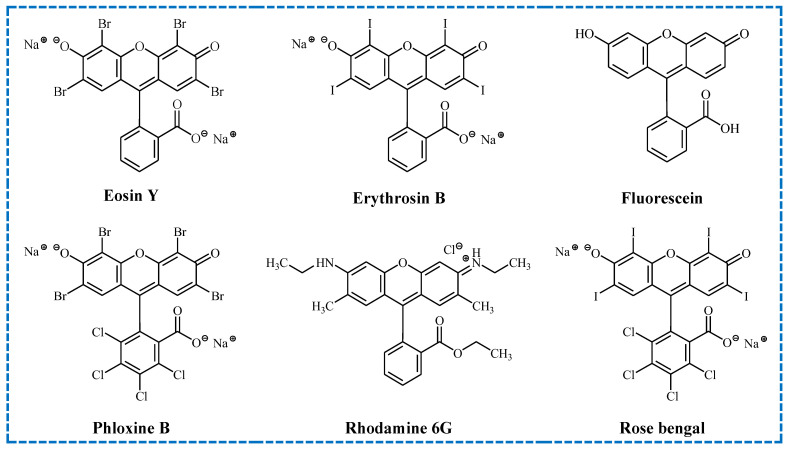
Chemical structures of organic chromophores used as photoactivators for PET-RAFT processes [92,95,96].

Among activators from the group of organic dyes used in photoinduced electron/energy transfer–reversible addition–fragmentation chain transfer polymerization, the following should be mentioned: eosin Y (EY), erythrosin B (EB), fluorescein (Fl), phloxine B (PB), rhodamine 6G (R6G), rose bengal (RB), etc. (Figure 9) [92,93,94,95,96].

For example, halogenated xanthene dyes (EY, EB, PB and RB) show a good ability to mediate in PET-RAFT processes. The degree of conversion of *N,N*-dimethylacrylamide (DMA) reached ca. 80%. Moreover, the use of these photocatalyst allowed for obtaining high kinetic parameters of polymerization, even in the presence of oxygen, which acts as an inhibitor and can terminate the growing polymer chain [96]. Similar efficiency of photocatalyst systems was described by Figg and colleagues [94]. The use of the combination eosin Y/2-(ethyl trithiocarbonate)-2-methylpropionic acid/(4-dimethylaminopyridine) in an appropriate molar ratio for radical polymerization of DMA results in monomer conversion of 25–95%.

As is well known, in the classical ATRP, the atom transfer step is crucial for the chain reaction to grow. Primarily used photocatalysts in atom transfer radical polymerization include metal-based compounds. However, the progress of research conducted in the area of controlled polymerization reactions has led to the use of metal-free systems as a good alternative for typical organometallic complexes [97]. Since then, this approach has become a guiding topic for the development of novel photoactivators. The mechanism of metal-free ATRP processes has been described in detail in numerous papers [83,97,98]. In short, the light-excited PC molecule causes the reduction of alkyl bromide by an oxidative quenching cycle. Then, a deactivating photocatalyst complex and propagating radical are formed. In the next step, the photocatalyst radical cation halide anion complex deactivates growing polymer chains. As a result, PC is regenerated and the polymer is end-capped by the bromide at the end of each cycle [98,99,100].

**Figure 10:**
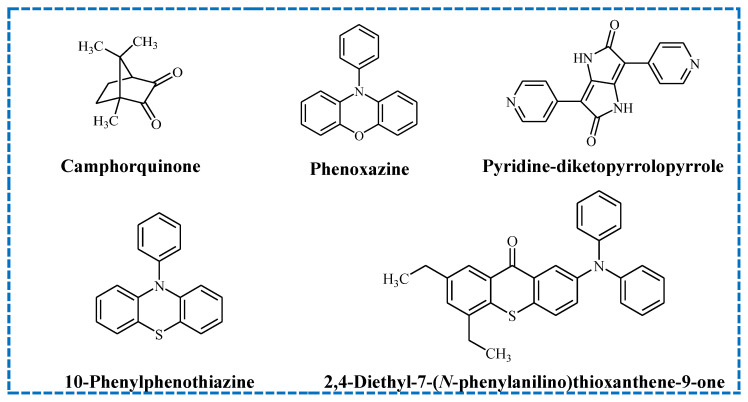
Chemical structures of organic dyes used as photocatalysts for metal-free ATRP processes [102,106,108].

It is worth emphasizing that many research papers describe the use of various organic dyes as metal-free ATRP activators, such as camphorquinone [101], diketopyrrolopyrrole derivatives [102,103], phenothiazines [83,97], phenoxazines [104,105], thioxanthones [106,107] and others [98,100]. Chemical structures of selected compounds are shown in Figure 10. For example, in 2020 Yang and colleagues [102] proposed a novel pyridine–diketopyrrolopyrrole (Figure 10) metal-free organophotoredox activator for the atom transfer radical polymerization of methyl methacrylate (MMA) and styrene. This photocatalyst system operated effectively in the visible-light spectrum and allowed researchers to obtain crosslinked polymers. Another interesting work in this field was presented by the Ortyl research group [106]. The scientists proposed novel mono- and disubstituted thioxanthones as photosensitizers for radical/cationic polymerization of acrylate/epoxy monomers. The synthesized dyes have been also used as photocatalysts for ATRP processes. The chemical structure of one of the most effective photosensitizer/photoactivators, i.e., 2,4-diethyl-7-(*N*-phenylanilino)thioxanthene-9-one, is depicted in Figure 10. The conversion of methyl methacrylate with the use of thioxanthone-based photocatalysts was in the range from 15% to 33%. Despite relatively low values of monomer conversion, the chromophores from the group of thioxanthenes seem to be promising candidates as photoactivators for metal-free atom transfer radical polymerization.

The above-mentioned examples are only a few achievements of recent years in the field of highly efficient dye-sensitized photoinitiating systems dedicated to radical (or ionic) polymerization reactions. The research works in this area aimed at searching for new compounds characterized by high activity under milder conditions than those which require the use of traditional irradiation sources with high power. An important aspect is also the designing of new dyes which show a negligible harmful impact on the natural environment [109].

## 4. Novel Series of Dye-Photosensitized Systems for Radical Polymerization Reactions

**Figure 11:**
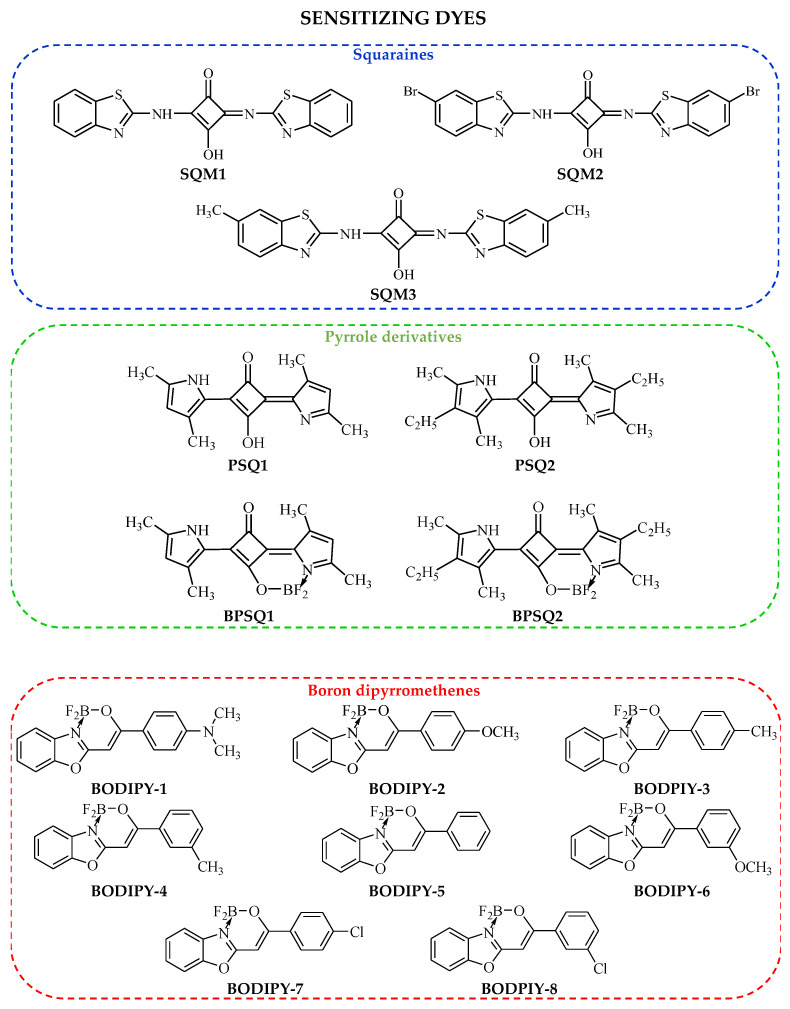
Chemical structures of sensitizers.

**Figure 12:**
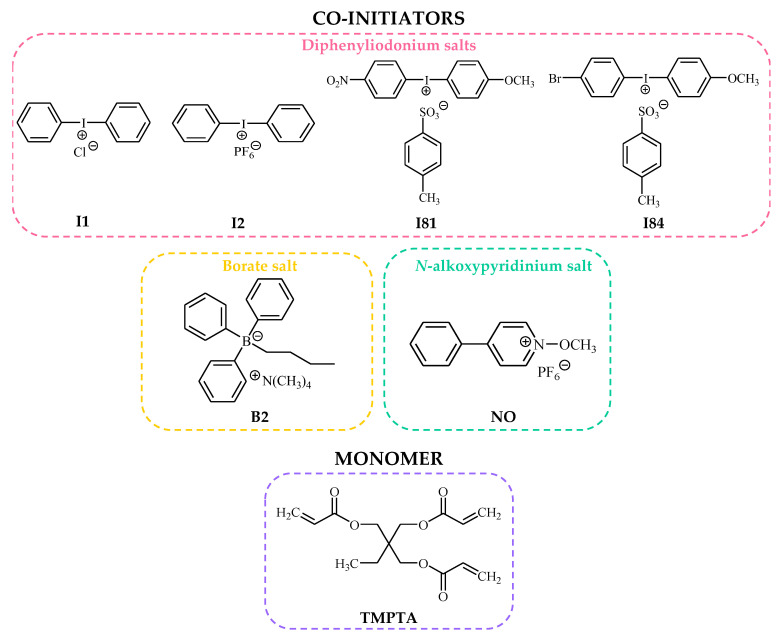
Chemical structures of co-initiators and monomer.

As mentioned in the literature, an excellent ability for sensitization of polymerization reactions is shown by the photoinitiating systems comprising squaraine, pyrrole or boron dipyrromethene derivatives [109,110,111,112]. Our research group contributed to the development of initiators based on this type of sensitizers, as described in several papers. Particularly, these achievements concerned two- and three-component PISs comprising squarylium dyes; however, we did not limit ourselves only to such examples [70]. The latest works published in 2021 and 2022 present new dyeing bimolecular photoinitiators for radical polymerization of acrylate monomers [113,114,115]. The chemical structures of the compounds included in these photoinitiating systems are presented in Figure 11 and Figure 12.

**Figure 13:**
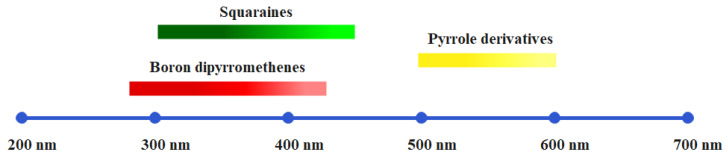
Absorption ranges of novel sensitizers.

The proposed systems were a combination of an appropriate sensitizer, i.e., squaraine dye (SQM1-SQM3), pyrrole derivative (PSQ1/PSQ2/BPSQ1/BPSQ2) or boron dipyrromethene (BODIPYs 1–8), with a selected co-initiator, such as diphenyliodonium salts (I1, I2, I81, I84), borate salt (B2) or *N*-alkoxypyridinium salt (NO). Depending on the chemical structure of the dye used, novel photoinitiators operate upon ultraviolet (UV), visible (Vis) or UV-Vis light, as shown in Figure 13.

The squaraines (SQM1-SQM3) absorb in the range from 300 nm to 450 nm. These compounds show activity in ultraviolet and visible light regions. On the other hand, the systems containing one of pyrrole derivatives (PSQ1, PSQ2, BPSQ1 or BPSQ2) are suitable for initiating the radical polymerization of various monomers in visible light. The absorption spectrum extend from 500 nm to 600 nm. The photoinitiators based on boron dipyrromethenes (BODIPYs 1–8) work under UV-Vis light. The absorption bands of BODIPYs 2–8 extend from 280 nm to ca. 410 nm and from 280 nm to 480 nm for BODIPY-1. The groups of new dyes described can be successfully used for sensitization of polymerization reactions and the light source can be readily adapted to the absorption characteristics of selected compounds.

As highlighted earlier, the panchromatic sensitization of polymerization needs the use of a suitable photoinitiating system containing dye molecules as a primary absorber and a second compound as the main initiator of chain reaction. These pairs of sensitizers/co-initiators should efficiently interact with each other to further the formation of radicals that will initiate polymerization process. It should be noted that most of the common initiators absorb over a narrow range of light, usually much below 300 nm. This is a significant difficulty in relation to the type of light sources most commonly used. However, this problem can be easily solved by the introduction of an appropriate sensitizer that will shift the sensitivity of PIS towards longer wavelengths. Therefore, the combination of initiators, such as diphenyliodonium salts, borates or *N*-alkoxypyridinium salts, with the proposed light absorbers (squaraines/pyrrole derivatives/boron dipyrromethenes) may act as active photoinitiators of radical polymerization. The proposed PIs show diversified photoinitiating abilities. The spectral characteristics of novel sensitizers, as well as the effectiveness of systems containing the mentioned dyes for initiation the radical polymerization of acrylates, are described in detail in this section.

### 4.1. Squaraine-Based Photoinitiators

**Figure 14:**
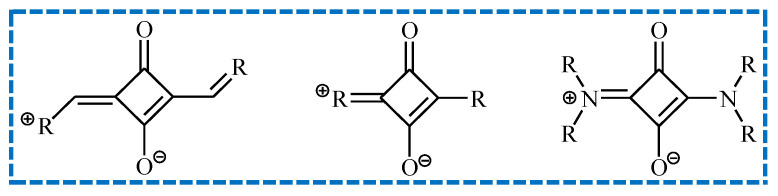
General structure of squaraine dyes.

Squaraines (SQs), known also as squarylium dyes, belong to the family of polymethines. Their characteristic feature is the donor–acceptor–donor (D-A-D)-type π-conjugated structure, in which the central unit is a four-membered ring derived from 3,4-dihydroxycyclobut-3-en-1,2-dion (called squaric acid) connected with two electron-donor groups. Depending on the type of substituents (R), symmetrical and unsymmetrical squaraines can be distinguished. Taking into account the chemical structure, there are three different types of squaraines, as presented in Figure 14 [116]. The basic synthetic route is the condensation reaction of 1 eqv. of squaric acid with 2 eqv. of systems containing electron-donating moieties [117,118,119,120].

The squaraines are characterized by excellent and unique physicochemical properties. It is primarily about strong absorption and emission in a wide range of the spectrum, even from UV to NIR. The values of molar extinction coefficients oscillate about 10^5^ M^−1^ cm^−1^. Moreover, these chromophore systems also tend to demonstrate high fluorescence quantum yields and biocompatibility as well as good photostability and photoconductivity [121,122].

**Figure 15:**
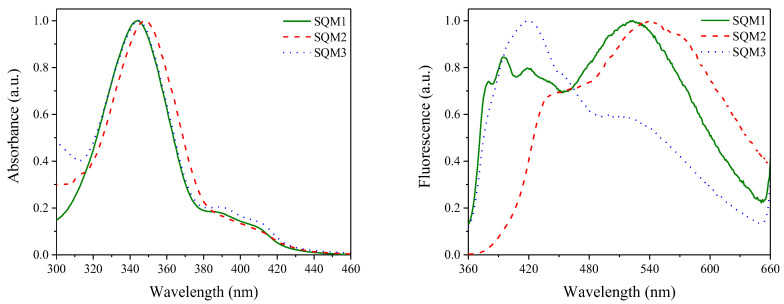
Normalized absorption (**L**) and fluorescence (**R**) spectra of squaraine dyes (SQM1-SQM3) registered in 1-methyl-2-pyrrolidinone (MP) at room temperature. Reproduced from ref. [113], which was published under a CC BY license.

As mentioned in Section 4, the squarylium dyes SQM1-SQM3 exhibit strong absorption in the UV-Vis region of spectrum. The absorption bands are narrow but strong, with the maximum located at about 345 nm. The values of molar extinction coefficients are in the range of 8.3 × 10^3^ M^−1^ × cm^−1^ to 2.5 × 10^4^ M^−1^ × cm^−1^. The fluorescence bands are relatively wide with two maxima and extend from 360 nm to 660 nm. The squaraines are also photobleached after exposure to argon ion laser radiation. The action of light with an intensity equal to 50 mW × cm^−2^ on the dye solution in *N,N*-dimethylformamide (DMF), both alone and in the presence of an appropriate co-initiator, results in a gradual decrease of the absorbance. Importantly, the steady-state photolysis experiments indicated that there are significant interactions between sensitizer and co-initiator. The spectroscopic characteristics of squaraine dyes are presented in Figure 15 [113].

**Figure 16:**
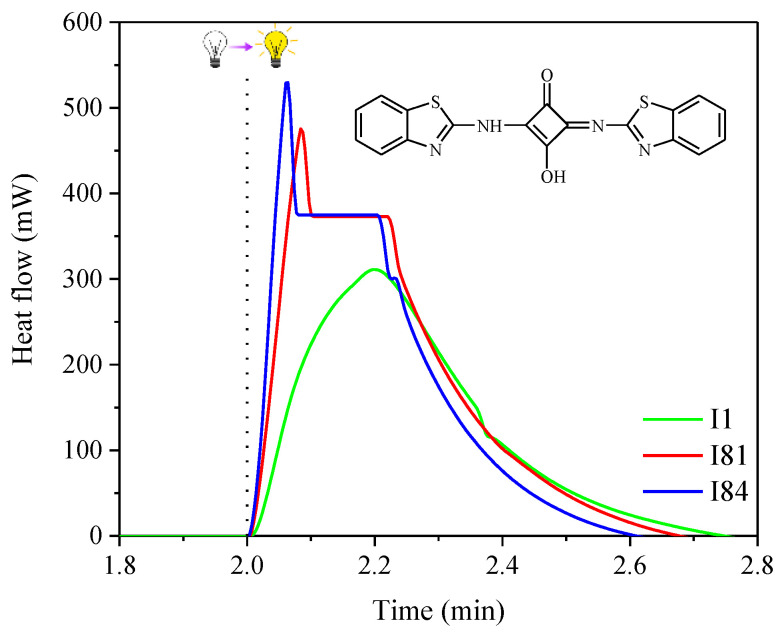
Kinetic profiles recorded during radical polymerization of trimethylolpropane triacrylate (TMPTA) initiated by two-component photoinitiating system consisting of SQM1 as sensitizer in the presence of various co-initiators, marked on the figure. Experimental conditions: concentration of PI—5 × 10^−3^ M; light intensity—30 mW × cm^−2^. Reproduced from ref. [113], which was published under a CC BY license.

The initiating efficiency of novel photoinitiators was determined by studying the kinetics of the radical polymerization of trimethylolpropane triacrylate (TMPTA). The experiments were carried out for two-component photoinitiating systems consisting of an appropriate sensitizer—1,3-*bis*(benzothiazolamino)squaraine (SQM1), 1,3-*bis*(6-bromobenzothiazolamino)squaraine (SQM2) or 1,3-*bis*(6-methylbenzothiazolamino)squaraine (SQM3)—in combination with one of the co-initiators, such as diphenyliodonium chloride (I1), (4-methoxyphenyl)-(4-nitrophenyl)iodonium *p*-toluenesulfonate (I81) or (3-bromophenyl)-(4-methoxyphenyl)iodonium *p*-toluenesulfonate (I84). The selected results are illustrated in Figure 16. As can be seen, irradiation of the polymerizable mixture causes an immediate reaction. The values of maximum heat flow ranged from 300 mW for SQM1/I1 to about 550 mW for others [113].

Taking into account the concentration of photoinitiator, the best kinetic parameters were shown by the combination of squaraine dye (SQM3) and iodonium salt (I81) in the amount of 5 × 10^−3^ M. The use of this photoinitiating system gave a value of conversion above 35% and photoinitiation index slightly above 5 × 10^−3^ s^−2^ [113].

The dyes from the family of squaraines are promising sensitizers, which in combination with iodonium salts form a high performance system initiating a photopolymerization reaction. Therefore, it can be concluded that the proposed photoinitiating systems effectively initiate the radical polymerization of acrylates in the UV-Vis light range. The described photoinitiators are distinguished by a short inhibition period and very short time of photocuring for the monomer composition, which does not exceed 60 s.

### 4.2. Photoinitiators Containing a Pyrrole Unit

**Figure 17:**
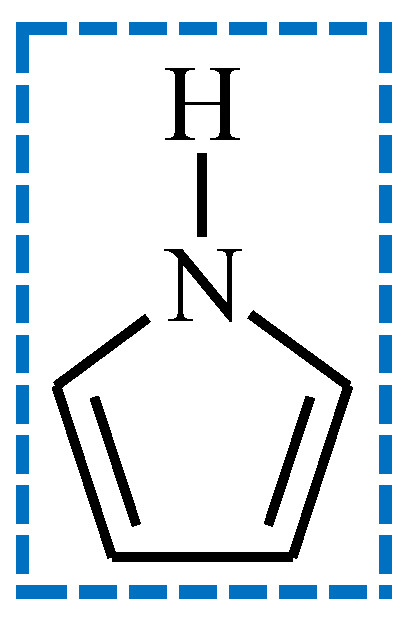
General structure of pyrrole-based dyes.

Recently, the dyes based on the pyrrole structure have become a substantial group of sensitizers used in photopolymerization reactions. Pyrrole (PY) is a five-membered heterocyclic ring compound containing a nitrogen atom with a lone pair of electrons, which causes this moiety to become an excellent electron-donating group [123,124]. The formula of a pyrrole moiety is depicted in Figure 17.

Many strategies for the synthesis of compounds based on the pyrrole structure can be found in the literature. The typical synthesis routes of PYs are described in detail in the work of Shi and others [125]. The characteristic feature of pyrrole dyes is a wide range of absorption, from ultraviolet to visible light, and high values of the ε parameter. These compounds are described in the literature mainly in terms of their applicability in medicine and pharmacy [126].

**Figure 18:**
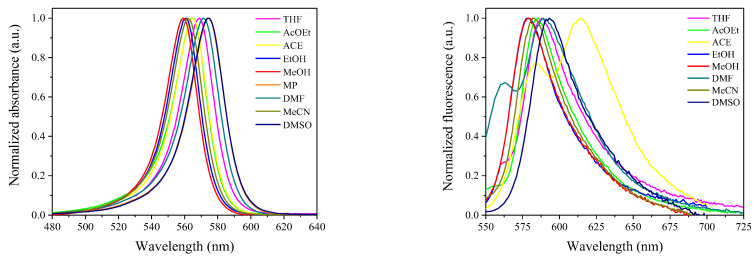
Normalized absorption (**L**) and fluorescence (**R**) spectra of BPSQ2 registered in solvents of various polarities at room temperature. Reproduced from ref. [114], with permission from The Royal Society of Chemistry, 2022.

Another group of dyes are chromophore systems containing two dimethylpyrrole moieties, such as 2,4-*bis*(3,5-dimethylpyrrol-2-yl)squaraine (PSQ1), 2,4-*bis*(4-ethyl-3,5-dimethylpyrrol-2-yl)squaraine (PSQ2), 2,4-*bis*(3,5-dimethylpyrrol-2-yl)squaraine difluoroborate (BPSQ1) and 2,4-*bis*(4-ethyl-3,5-dimethylpyrrol-2-yl)squaraine difluoroborate (BPSQ2). In general, these compounds can be classified into the group of squaraines, wherein BPSQ1 and BPSQ2 are their difluoroborate complexes. The described dyes absorb in narrow range of the spectrum, from about 500 nm to 600 nm. The values of molar extinction coefficients achieved 10^5^ M^−1^ cm^−2^. The fluorescence bands are wide. These compounds emit light in the Vis region, from 550 nm to 700 nm. The fluorescence quantum yields (φ_fl_) were at the level of 10^−3^ and the average value of the transition energy from the ground to the excited state (E_00_) achieved 2.20 eV. All of squaraines undergo a photodegradation process in the presence of co-initiator molecules during irradiation at 514 nm. The selected absorption and fluorescence spectra of pyrrole derivatives are presented in Figure 18 [114].

**Figure 19:**
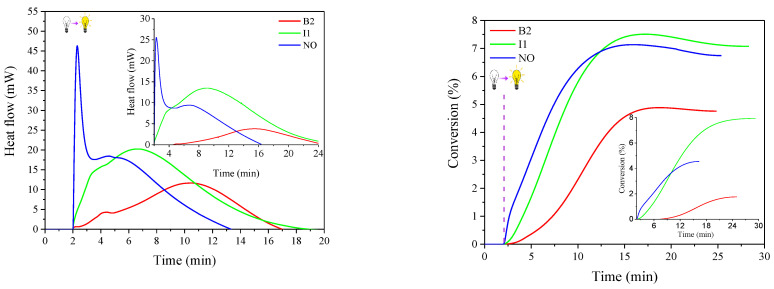
Kinetic profiles recorded during radical polymerization of trimethylolpropane triacrylate (TMPTA) initiated by two-component photoinitiating system consisted of PSQ1 (or PSQ2—inset) as sensitizer in the presence of various co-initiators, marked on the figure. The experimental conditions: concentration of PI—1 × 10^−3^ M; light intensity—50 mW × cm^−2^. Reproduced from ref. [114], with permission from The Royal Society of Chemistry, 2022.

The proposed sensitizers in combination with a suitable co-initiator, i.e., tetramethylammonium *n*-butyltriphenyl borate (B2), diphenyliodonium chloride (I1) or 1-methoxy-4-phenylpyridinium tetrafluoroborate (NO), give a highly active photoinitiating system for radical polymerization of trimethylolpropane triacrylate (TMPTA), as shown in Figure 19. The most effective PISs contained 1 × 10^−3^ M of photoinitiator. It has been found that further increasing of the amount of initiator decreases the rate of polymerization, what may be explained by the inner filter effect. It means that a high concentration of a strongly absorbing dye molecule decreases the penetration of light into deeper layers of the polymerizable formulation, which causes a slowing down or total inhibition of the polymerization reaction.

As is clearly seen in Figure 19, the type of co-initiator used has a major effect on the kinetics of polymerization. Generally, the slowest are photoinitiating systems containing borate salt (B2). On the other hand, the best efficiency was shown by compositions with *N*-alkoxypyridinium salt (NO). The maximum degree of conversion (C_%_ = 12.4%) was obtained for PSQ1/NO initiator, whereas the highest value of polymerization rate (R_p_ = 43.10 × 10^−4^ s^−1^) was observed for a PSQ2/NO combination. The photoinitiation indices ranged from 1.38 × 10^−7^ s^−2^ to 317.85 × 10^−7^ s^−2^. On the basis of the experiments conducted, the photoinitiation mechanism of the radical polymerization reaction was developed. It turns out that a key role in radical formation can be attributed to the photoinduced electron transfer (PET) between sensitizer and co-initiator molecules. Two onium salts act as the electron acceptors, whereas borate salt is an electron donor. The photoinitiating abilities of novel systems may result from the reactivity of the initiating radicals formed by PET: *n*-butyl, phenyl and methoxy for B2, I1 and NO, respectively [114].

To conclude, the photoinitiating systems composed of sensitizers based on pyrrole rings effectively initiate the radical polymerization of acrylates. Because of beneficial properties of these dyes, such as high molar extinction coefficients and wide ranges of absorption, pyrrole derivatives in combination with a suitable co-initiator can be successfully used as highly efficient activators of polymerization processes.

### 4.3. BODIPY-Based Photoinitiators

**Figure 20:**
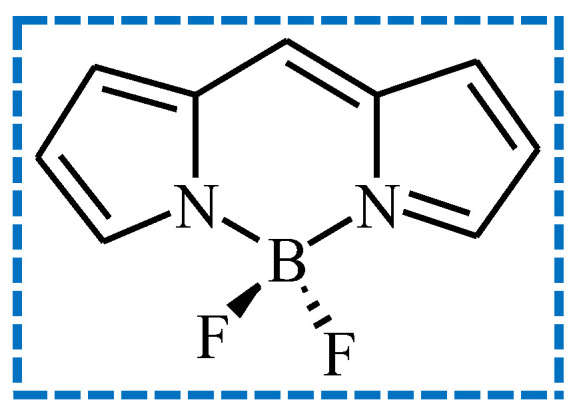
General structure of boron dipyrromethene dyes.

Boron dipyrromethenes (BODIPYs), the full name of which is 4,4-difluoro-4-bora-3a 4a-diaza-*s*-indacenes, are an important group of functional dyes used in different areas of science and technology. The characteristic structural feature of these compounds is the presence of a BF_2_ moiety connected to a dipyrromethene core. The general formula for this group of dyes is presented in Figure 20.

Because of the significant popularity of these compounds, many methods for their synthesis have been developed by this time. A detailed overview of the directions of synthesis can be found in the paper by Bumagina and colleagues [127]. The BODIPYs show strong absorption and emission, high fluorescence quantum yields, good thermal and photochemical stability and low phototoxicity. These features make them candidates for uses in photonics, bioimaging, organic dye-sensitized solar cells and as optical chemosensors, etc. [128,129,130].

**Figure 21:**
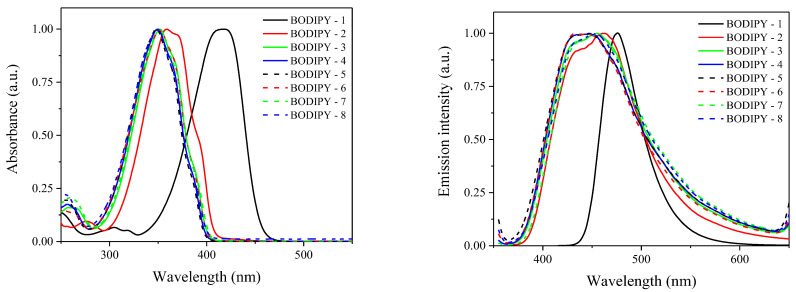
Normalized absorption (**L**) and fluorescence (**R**) spectra of boron dipyrromethene dyes (BODIPY-1–BODIPY-8) registered in acetonitrile at room temperature. Reproduced from ref. [115], which was published under a CC BY license.

The newly designed dyes belonging to the group of boron dipyrromethenes are 2-phenacylbenzoxazole difluoroboranes, differing from each other in the type of substituent on the C3 and C4 atoms of the phenyl ring (Figure 11). These dyes show strong absorption and emission in the range of UV-Vis light, which is depicted in Figure 21. The maximum of absorption is located at about 350 nm. Moreover, the values of molar extinction coefficients are in the range from 2.3 × 10^4^ M^−1^ × cm^−1^ to 4.3 × 10^4^ M^−1^ × cm^−1^. The Stokes shifts for all derivatives were similar, about 6 × 10^3^ cm^−1^, and the fluorescence quantum yields oscillate from 0.48 × 10^−2^ for BODIPY-4 to 0.98 for BODIPY-1.

**Figure 22:**
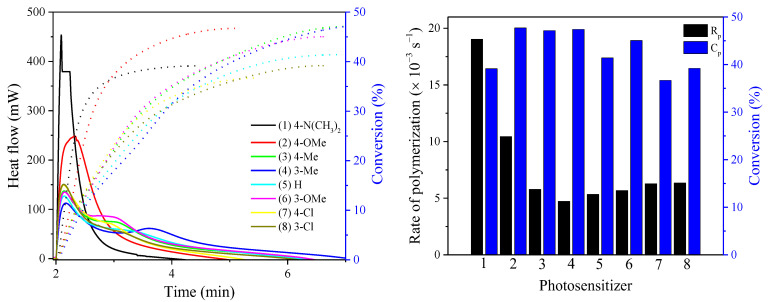
Kinetic profiles recorded during radical polymerization of trimethylolpropane triacrylate (TMPTA) initiated by two-component photoinitiating systems consisting of BODIPY dye as sensitizer (marked on the figure) in the presence of 1-methoxy-4-phenylpyridinium tetrafluoroborate (NO). The experimental conditions: concentration of PI—1 × 10^−3^ M/1 × 10^−2^ M (sensitizer/co-initiator); light intensity—50 mW × cm^−2^ (solid line denotes data of heat flow during polymerization; dash line refers to monomer conversion). Reproduced from ref. [115], which was published under a CC BY license.

The photoinitiating abilities of new systems composed of an appropriate boron dipyrromethene derivative with the combination of co-initiator, such as tetramethylammonium *n*-butyltriphenyl borate (B2), diphenyliodonium hexafluorophosphate (I2) and 1-methoxy-4-phenylpyridinium tetrafluoroborate (NO), were examined and compared with commercially available initiators. The selected results are presented in Figure 22.

From the kinetic curves, one can conclude that all photoinitiating systems show good activity. The degree of monomer conversion (C_%_), as well as the rate of polymerization, depend on the type of sensitizer and co-initiator, their concentration in the polymerizable mixture and on the intensity of light used in experiments. The values of C_%_ ranged from ca. 20% for the PISs containing BODIPY dye (1–8) and iodonium salt (I2) to 50% for BODIPY dye/*N*-alkoxypyridinium salt (NO). The rate of polymerization was on the level of 2 × 10^−3^ s^−1^ to about 20 × 10^−3^ s^−1^. It has been also observed that a light intensity of 10 mW × cm^−2^ is sufficient for effective photoinitiation. Further increases in light intensity do not improve the kinetic parameters of the polymerization reaction. Compared to commercial initiators, such as camphorquinone/*N*-phenylglycine, the most effective system—BODIPY-1/NO—shows better photoinitiation properties. Interestingly, the final polymer probes initiated by photoinitiating systems containing 2-phenacylbenzoxazole difluoroboranes and borate/iodonium salt/pyridinium salt were photoluminescent. This feature provides for the possibility of application in strongly photoluminescent layers and coatings [115].

The use of boron dipyrromethenes in photopolymerization reaction is valid due to good absorption properties and high activity of sensitization of radical polymerization reactions under mild conditions. Moreover, the unique features of these compounds provides for the opportunity to use them in areas with special requirements, such as medicine. Therefore, the desire to expand this group of color compounds seems to be fully justified.

### 4.4. Summary of the Effectiveness of the New Dye-Based Photoinitiating Systems

**Figure 23:**
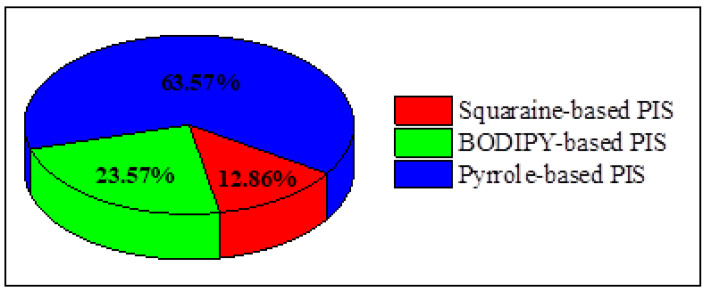
Diagram illustrating the relationship of the number of research papers in 2012–2022 on dyeing photoinitiating systems depending on the type of used photosensitizer. Based on data from Science Direct. Keywords: squaraine AND photoinitiator, BODIPY AND photoinitiator, pyrrole AND photoinitiator. Accessed on 13 February 2023.

As mentioned above, there are many papers focused on dye-based photoinitiating systems. Due to the variety of organic dyes, the authors of research publications propose various combinations of sensitizer/co-initiator systems. Based on the data obtained from Science Direct, we investigated the contribution of dyeing photoinitiators based on squaraines, BODIPYs and pyrrole derivatives. In the years 2012–2022, the number of works on the topic of dyeing PISs were 18, 33 and 84 for photoinitiating systems containing squaraine (SQ), boron dipyrromethene (BODIPY) and pyrrole (PY) as photosensitizers, respectively. The comparison of number of documents published in each PIS is presented in Figure 23.

To summarize, all of the described dyes show excellent spectral properties for use as sensitizers in PISs for the radical polymerization of acrylate monomers. All squaraines, pyrrole derivatives and boron dipyrromethenes are good candidates for use as components of dye-based photoinitiating systems. Table 1 summarizes the kinetic parameters of the photopolymerization process for acrylate monomer (TMPTA) obtained through the use of bimolecular colored photoinitiators.

As can be clearly seen in Table 1, the proposed photosensitizers absorb light in the UV-Vis range. All of synthesized dyes exhibit similar spectroscopic properties and molar extinction coefficients of about 10^4^ M^−1^ × cm^−1^. A simple modification of the structure, consisting of the introduction of an appropriate substituent to the chromophore moiety—such as an electron-pushing or an electron-withdrawing group—allows the physicochemical properties of this dye to be changed. It also allows for fine-tuning of absorption to the light source used in photopolymerization experiments. The proposed photoinitiators act in mild radiation conditions, which is important from an economic and environmental point of view. The use of photoinitiating systems under study provides high kinetic parameters of the polymerization process, i.e., C_%_ ≈ 10–50% and a rate of reaction (R_p_) from 0.2 × 10^−3^ s^−1^ to 26 × 10^−3^ s^−1^.

## 5. Requirements and Challenges for Radical Photoinitiators

The wide diversity of commercially available initiators provides a large range of absorption profiles which cover the whole spectrum of ultraviolet (UV) and visible (Vis) light. However, due to increasing requirements for new photoinitiators, the search for new systems for highly efficient photopolymerization reactions is extremely important.

The basic guidelines for photoinitiators include good physico-chemical stability, high initiation quantum efficiency, compatibility of absorption spectrum with the characteristics of a light source, non-toxicity for the environment and humans, good solubility in polymerizable composition and zero negative impact of the final product. It is also very important that new initiators are highly effective at low concentrations and low light intensity (primarily when using visible light or LED). Most of these requirements are feasible, but with the development of science and technology new problems and new challenges appear [114,131].

The development of novel procedures to reduce the problem of the environmental impacts of polymerization will allow for a change in the perception of the industry as dangerous for humans and nature. Future perspectives will focus primarily on the obtaining of PISs from scaffolds of natural products. These solutions have been already proposed by Lalevée and colleagues, who presented the possibility of obtaining chalcones from plants and their use in the photopolymerization for the production of hydrogel materials [132]. Likewise, Dumur and others described an overview of the various visible light photoinitiators based on natural products [133]. In addition, it is also worth mentioning here another promising possibility, that of quantum photoinitiators. One of the newest literature reports presents modern and promising nanocrystal PIs [134]. These compounds are competitive with the typical organic analogues and can play the role of a specific photocatalysts, e.g., for the production of various polymer materials for medical applications. As of yet, it has been shown that quantum initiators show low migration, tunable blue excitation, high efficiency in aqueous media and low energy activation. Certainly, further works in this field will provide more information on the mechanism of action for this new group of photoinitiators [134].

## 6. Conclusions

In this review, we summarized the newest advances in the design of high-performance photoinitiators. The photoinitiating systems described are dedicated to radical polymerization reactions, mainly of acrylate monomers. The activation of polymerization reactions occurs as a result of the interaction of the dye with a suitable co-initiator, such as borate, iodonium or *N*-alkoxypyridinium salt. The use of these photoinitiators provides for monomer conversion in a range from 10% to about 50% and high rates of polymerization, ca. 1 × 10^−2^ s^−1^. The proposed combinations of sensitizer/co-initiator show high performance in UV-Vis range of the spectrum.

Taking into account the limited absorption range of some sensitizers, future works will focus on developing novel light absorbers active in a wider range of the spectrum. An appropriate modification of the structure of these compounds may improve their spectroscopic properties and thus enable better light penetration within polymerizable compositions. Certainly, particular attention should be paid toward panchromatic and quantum photoinitiators. Moreover, human and environmental safety must also be taken into account when designing new compounds.

## Figures and Tables

**Table 1:** Summary of the spectroscopic properties of photosensitizers and the kinetic parameters of the radical polymerization of TMPTA.

Sensitizer	λ_max_ (nm)	ε (× 10^4^ M^−1^ cm^−1^)	Light Intensity (mW cm^−2^)	R_p_ (× 10^−3^ s^−1^)	C_%_ (%)
SQM1	344	2.45	30	20.00	29.4
SQM2	348	0.97		25.80	29.7
SQM3	345	0.83		23.40	35.7
PSQ1	553	5.90	50	0.26	12.4
PSQ2	562	8.40		0.36	12.0
BPSQ1	553	7.70		1.25	6.3
BPSQ2	562	11.00		0.56	10.7
BODIPY-1	415	4.25	50	19.04	39.2
BODIPY-2	359	3.29		10.45	47.7
BODIPY-3	351	3.12		5.78	47.1
BODIPY-4	350	2.30		4.73	47.4
BODIPY-5	349	2.70		5.34	41.4
BODIPY-6	351	2.67		5.68	45.1
BODIPY-7	352	2.96		6.28	36.7
BODIPY-8	350	2.96		6.34	39.2

## Data Availability

The data presented in this study are available on request from the corresponding author.
